# Criticisms on the Treatment of the Venereal Disease

**Published:** 1802-06

**Authors:** T. Vage


### *Criticisms on the Treatment of the Venereal Disease,*

By T. Vage, M. D. F. R S.

[ Continued from Vol. III. p. 465—471. ]

AGREEABLY to promife, in a former Paper upon the local and recent fymptoms of the Venereal Difeafe, I now fend you a few Remarks upon the confirmed ftate of it, and upon the infirmities of conftitution which frequently remain after its cure. In ftepping forward on fubjects which have already undergone the difquifitions of men of the firft abilities, I can only obferve, that a very extenfive experience in military hofpitals for many years, has clearly evinced that the ufual modes of treatment, in thefe refpects, ftill continue very defective. But while that experience fhewed the infufficiency of practice, it was confidered, at the fame time, to afford ample opportunities of improvement, by admitting of rational deviations; and the refults of a multiplicity of trials in fuch cafes, as far as they were found ufeful, are here fubmitted to your confideration. From the feveral ingenious theories and fedulous refearches of many eminent practitioners to advance medical knowledge, in this and other diforders, and which at prefent emuloufly engage their attention, we may expect much information; in the mean time, a little practically afcertained may probably be acceptable.

The abforption of the venereal virus into the circulation, which may be termed its fecondary infection, generally contaminates the habit to an extenfive degree before it fhews any figns of its prefence; and hence, when it does appear, its fymptoms are far more fevere and obftinate than thofe of its incipient infection, although they arife from fimilar fources, from from the inflammation and ulceration of the folids. It is a fingular circumftance of the venereal poifon, that fuch a depravation of the fluids as it here produces can exift for a length of time, without difturbing the conftitution in any remarkable degree. Some parts of the fyftem appear more fufceptible of the infection than others, as the glands, membranes, bones, periofteum, cartilages, &c. and hence nocturnal pains in various parts of the body, violent head-achs, defedations and ulcerations of the fkin, and the like.* As this difeafe has, naturally, no limits to its progrefs, it invades by degrees all the functions of the animal economy, imitating a variety of other complaints, as afthma, phthifis, paralyfis, dyfpepfia, hypochondriafis, &c. until at length, after a feries of the moft excruciating torments, the whole frame perifhes under its ravages, the moft ghaftly fpectacle of human wretchednefs.

The venereal infection would appear to be at firft communicated in different degrees of virulence. In fome it is only a flight gonorrhœa, with fcarce any inflammation; in others, it attacks the parts with which it comes in contact at once with ulceration, as chancres and fevere urethral corrofions; and in proportion to this firft mildnefs or acrimony, when it gets into the habit, it is flower or fpeedier in the production of its fecondary fymptoms. I have met with feveral cafes of confirmed lues, which required fome years to become fo, where the incipient infection was flight; while it is common to fee the fymptoms of a general contamination in the fpace of a few months from that degree of virulence which produces chancre. It has been already obferved, contrary to the opinions of fome, that the fooner the recent or local ulcerations are cured, the better, on account of the abforption of the infectious matter, which in all cafes, in fome degree or other, takes place; yet it muft be further obferved, that nothing is more deceitful than a fpeedy cure of all venereal fores, although fo neceffary; as it is apt to prompt to a hafty conclufion, that every thing is accomplifhed, and which I am forry to fay, feems frequent with many practitioners, to acquire the repute of expertnefs. The ceffation, therefore, of all venereal appearances, fhould be followed for fome time with the correcting alteratives.

The infectious particles themfelves perhaps fuffer no change, and only become more or lefs virulent in being more or lefs concentrated. For certainly, the more numerous the points of infection are in any part, the greater the corrofion will be. And

* Several parts which Haller fuppofes deftitute of fenfation, become exquifitely fenfible in a ftate of inflammation.

And what particularly accelerates that corrosion, is, that the infectious matter is generated by the corruption, and not saturated like the phlegmonic acrimony of some abscesses. The external mode of action of the venereal poison explicitly teaches us its internal operations; but all its internal attacks may be regarded as so many sources, which propagate the infection more effectually, in being removed from inspection, and the direct application of remedy. Another thing that frequently promotes the progress of the disease, is a neglect of its symptoms, from the patient's ignorance of their nature and presence, so that a considerable time elapses before any application is made for relief. But should the first symptoms be attended to, they ought not to be rated according to any apparent mildness, but from the importance of the parts attacked; for should these parts be essentially necessary to health, or contiguous to some which are so, a very little time may render the inflammation and ulceration extremely dangerous, as the virus is unremitting and speedy in its effects.

Great improvements in the treatment of recent infection, have been made within a few years of the last century; sorry am I to observe, that the endeavours of many eminent men have not been attended with similar success in the confirmed stages of it. The warm controversy concerning some new discoveries which still subsists between several gentlemen of much ingenuity, experience, and respectability, can afford but little assistance to those practitioners, who with prudence and safety conduct themselves by established precepts. Mercury is the only remedy as yet admitted by all as a specific for this disease; and yet in the most judicious use of it, disadvantages it has sufficiently momentous to warrant further researches, either for the prevention of its detriment to the habit, or for other specifics with less injurious properties. Among what are termed new discoveries, the nitric acid seems to claim the chief regard; for although it is not adequate to a perfect cure in confirmed cases, yet if given at proper intervals it very much prevents some of the ill effects of mercury, particularly upon the teeth and gums, and the debility of the digestive viscera. From these properties of the acid, it was reasonable to suppose that the mineral in combination with it, would improve in its medicinal efficacy; and from many trials of it as an alterative, I have had the satisfaction to find my expectations answered, not only in venereal complaints, but in scrofulous and cancerous cases also.

How far the substances by which mercury is divided, pass with it into the system, would seem a curious and perhaps a useful inquiry. It is capable in its simple state, however, when minutely divided, of mixing with the fluids, and of pervading the remoteſt receſſes of the ſyſtem. But as it is a ſubſtance incapable of aſſimilation, it muſt of courſe return from thence, to be evacuated from the habit by ſuch channels as are appointed to ſeparate the recrementitious parts of the circulation. The curative efficacy of mercury in venereal complaints, evidently ariſes from the correction of ſyphilitic virus, whether it appears in the inflammation or ulceration of the ſolid parts, or in the inquination of the fluids. The infectious matter propagates itſelf by the corruption, or, in other words, by changing the parts which it attacks into its own nature. Hence it is that the ſmalleſt ſpeck or particle of infection in any part, becomes rapidly progreſſive, even though there ſhould be no general taint in the habit, nor any of the original infection remaining. Nothing, therefore, can arreſt the progreſs of the diſeaſe but a change in the infectious acrimony, wherever it may exiſt; and there ſeems no other rational mode of effecting this but upon the principle of attractive affinity or combination, by which it is well known that ſubſtances loſe their original qualities, and for which mercury, by a minute diviſion, and its general diſpoſition to unite with other ſubſtances, is well adapted. That mercury is capable of acting in this manner, is further evident from its power of reducing ſome foul ulcers to a good complexion, whoſe acrimony is locally generated, and which would otherwiſe continue to be eroſive. As the mineral, then, cannot itſelf remain in the habit, it muſt conſequently carry with it the infectious matter, in combination, and thus rendered inert, to be evacuated by ſome of the emunctorial outlets. The internal inflamed and ulcerated parts, thus liberated from the corroſive matter, will, like external ones, aſſume a healing condition, if the ſolids are not much impaired, or the humours not much impoveriſhed.

This brings me to obſerve, that in courſes of mercury, which are requiſite for the cure of confirmed lues, nothing claims more attention than the ſupport of the digeſtive viſcera, becauſe a nutritious chylification is abſolutely neceſſary to enable Nature to repair the damages of ulcerated parts, while remedies correct the acrimony; and eſpecially as mercury has a direct tendency to debilitate theſe organs. For it is very obſervable, that even a common ſore will put on a bad complexion, merely from poor living, or a dyſpeptic habit. This precept ſo neceſſary, is however ſo little regarded, that it is no uncommon thing to ſee ulcers remain unhealed, after a ſtrict exhibition of mercury, and after the infection has been completely ſubdued. In whatever way then the mercury is uſed, it will be found of the utmoſt benefit to give ſtomachic corroborating bitters at proper intervals. It is upon this principle, perhaps, that that modern practice prefers the introduction of the fpecific by inunction. But, with full affent to this, I would ftill advife fome gentle ufe of it internally; efpecially as it can be done with fafety, in the moft delicate conftitutions, under the caution juft mentioned. For in a general contamination of the habit, the glands, as before remarked, are particularly fubject to the infection, and, of courfe, the glands of the mefentery, through which the lacteals pafs, cannot well efcape, and which, in fuch a cafe, muft vitiate the chyle. To compenfate for this, thefe glands are doubly within the power of remedy; firft in the general method of cure, and ftill more directly by the conveyance of the lacteals themfelves.

Under thefe precautionary ideas the habit muft be kept replete with the fpecific, until the difeafe is perfectly eradicated. This will require, at proper intervals, a repetition of inunction, or of whatever mode of ufing it may be adopted. For part of the remedy, which has pervaded the conftitution is conftantly paffing off through the emunctorial paffages. And here it requires fome addrefs to prevent the mercury, in fuch a degree of it, from difcharging itfelf by falivation, which, from fome failures of cure in my own practice, and from that of fome of the ableft practitioners, there is much reafon to fuppofe this evacuation uncertain, and in fome cafes inadequate to the purpofe. Any forced evacuation indeed prevents the remedy from pervading the fyftem fufficiently. A common cathartic, we fee, produces this effect, and even diverts its natural tendency to the falival glands. Befides thefe objections, fuch a conftant flux of humours to the head frequently injures the brain and origin of the nerves fo much, as greatly conduces to that debility of habit which often remains after cure, and which is always difficult to remove. Thefe and other reafons of the fame nature it was which probably induced fome to adopt, at firft, an alterative plan of treatment; but the modes which are generally adopted for this end, are not lefs objectionable than the former. Some give alterative dofes of the fpecific, indeed, but fubjoin cathartics at ftated times, which moft certainly counteract the falutary effects of the mercury. Others employ it in like manner, but without any regard to confinement or regimen, in confequence of which the remedy is either turned upon the bowels, and fruftrates the intention as much as if a cathartic was annexed to it; or elfe produces a fpitting, which requires cathartics to fupprefs; while others, again, unite the mercury with laxatives, which keep up nearly a continual diarrhœa. Mercury, therefore, can only act its part properly when it difturbs no function, and produces, or is accompanied with, no fenfible or copious evacuation; and the only outlet for this is

 through through the cuticular pores. Every point of the whole furface of the body is here employed for the purpofe; fo that an equal quantity, or more, may be difcharged this way than by any other emunctory, without any inconvenience. But the chief advantage is, that the remedy is never difcharged this way, until it has pervaded the habit, and confequently exerted its antivenereal qualities with proper extent and effect. And, indeed, the cuticular pores, upon another account, would appear the moft eligible for this purpofe, as they are the mode of evacuation which Nature generally appoints for the morbific matter in other difeafes. But the proper management of this bufinefs, it muft be confeffed, requires as much care, method, and regularity as falivation itfelf; the fame confinement, warm clothing, particularly the ufe of flannel; yet it will make ample amends for thefe inconveniences, in its certainty of fuccefs, in its being lefs hurtful to the conftitution, and generally in requiring lefs time.

[ To be continued. ]

